# AI-based structure-function correlation in age-related macular degeneration

**DOI:** 10.1038/s41433-021-01503-3

**Published:** 2021-03-25

**Authors:** Leon von der Emde, Maximilian Pfau, Frank G. Holz, Monika Fleckenstein, Karsten Kortuem, Pearse A. Keane, Daniel L. Rubin, Steffen Schmitz-Valckenberg

**Affiliations:** 1 Department of Ophthalmology, University of Bonn, Bonn, Germany; 2 Department of Biomedical Data Science, Radiology, and Medicine, Stanford University, Stanford, CA USA; 3 John A. Moran Eye Center, University of Utah, Salt Lake City, UT USA; 4 Augenklinik, Universität Ulm, Ulm, Deutschland; 5 Augenarztpraxis Dres. Kortüm, Ludwigsburg, Deutschland; 6 Moorfields Eye Hospital NHS Foundation Trust, London, UK

**Keywords:** Prognostic markers, Outcomes research

## Abstract

Sensitive and robust outcome measures of retinal function are pivotal for clinical trials in age-related macular degeneration (AMD). A recent development is the implementation of artificial intelligence (AI) to infer results of psychophysical examinations based on findings derived from multimodal imaging. We conducted a review of the current literature referenced in PubMed and Web of Science among others with the keywords ‘artificial intelligence’ and ‘machine learning’ in combination with ‘perimetry’, ‘best-corrected visual acuity (BCVA)’, ‘retinal function’ and ‘age-related macular degeneration’. So far AI-based structure-function correlations have been applied to infer conventional visual field, fundus-controlled perimetry, and electroretinography data, as well as BCVA, and patient-reported outcome measures (PROM). In neovascular AMD, inference of BCVA (hereafter termed inferred BCVA) can estimate BCVA results with a root mean squared error of ~7–11 letters, which is comparable to the accuracy of actual visual acuity assessment. Further, AI-based structure-function correlation can successfully infer fundus-controlled perimetry (FCP) results both for mesopic as well as dark-adapted (DA) cyan and red testing (hereafter termed inferred sensitivity). Accuracy of inferred sensitivity can be augmented by adding short FCP examinations and reach mean absolute errors (MAE) of ~3–5 dB for mesopic, DA cyan and DA red testing. Inferred BCVA, and inferred retinal sensitivity, based on multimodal imaging, may be considered as a quasi-functional surrogate endpoint for future interventional clinical trials in the future.

## 摘要

对于年龄相关性黄斑变性(AMD)的临床试验来说, 敏感且强大的视网膜功能测量手段是至关重要的。近期的新进展是利用人工智能(AI)来解释多模式影像成像的心理物理检查结果。我们以“人工智能”和“机器学习”为关键词, 结合“视野测量”、“最佳矫正视力(BCVA)”、“视网膜功能”和“年龄相关性黄斑变性”, 对PubMed和Web of Science中的文献进行了综述。到目前为止, 基于人工智能的结构功能相关性的研究已被应用于传统的视野检查、FCP视野检查、视网膜电生理检查以及BCVA的检查和患者报告结果(PROM)的测量。对于新生血管性AMD, BCVA推理法(以下称为BCVA推测法)可以评估BCVA结果, 其均方根误差大约为7-11个字母, 这与实际视力评估的准确性相当。此外, 基于人工智能的结构功能相关性的研究可以成功推测FCP视野检查的结果, 包括中间视觉、暗适应(DA)青色和红色试验(以下称为灵敏度推测法)。通过增加短时间的FCP检查, 可以提高灵敏度推测法的准确性, 对于中间视觉法、暗适应青色法和暗适应红色法, 其平均绝对误差(MAE)约为3-5 dB。基于多模态成像的BCVA推测法和视网膜灵敏度推测法, 可被认为是未来干预性临床试验的功能评估手段的替代终点。

## Introduction

Age-related macular degeneration (AMD) is the leading cause of visual disability among the elderly in industrialised countries [1]. While anti-vascular endothelial growth factor (VEGF) therapy has markedly improved outcomes for macular neovascularisation secondary to AMD, disease-specific therapy for early and non-exudative manifestations, including geographic atrophy (GA), is lacking [2, 3]. Beyond analysis of structural changes, there is an unmet need to establish meaningful functional endpoints for assessment of visual impairment associated with AMD manifestations. Best-corrected visual acuity (BCVA), the most common used functional endpoint, only measures photopic function of the central retina and is therefore not sensitive to measure therapeutic benefits outside of the fovea [4, 5]. Other measures, including low-luminance visual acuity (LLVA), reading speed, fundus-controlled microperimetry (FCP), and patient-reported outcome measures (PROM), potentially assess impairment of visual function in more detail. However, most of these tests are time consuming and can be particularly demanding for the elderly patient [6–9]. Therefore, alternative analysis strategies would be desirable that are more practicable and easier to obtain, while addressing different facets of visual function.

Artificial intelligence (AI) is creating a paradigm shift in every sector of medicine. Ophthalmology is at the forefront of implementing AI-enabled health care, with a few commercially available AI tools already available for clinical care. First applications have already crossed the threshold into clinical care [10]. These tools can now assist clinicians in diagnosis of fundus photographs as well as achieve automated annotations of optical coherence tomography (OCT) imaging [11–14]. In AMD, AI has been deployed to estimate the number of anti-VEGF injections needed and to predict GA progression [15, 16]. There is a wide range of potential AI-algorithms used for predictive modelling. In recent years, convolutional neural networks have proven particularly successful for image classification tasks by extracting features from raw data through hierarchies of increasing abstraction which superficially represent visual processing in the brain [17, 18]. These deep-learning (DL) algorithms require large image data sets and results can be hard to interpret. Machine-learning (ML) algorithms using pre-defined, “hand-crafted” (e.g. Random Forest or Lasso regression) can therefore be a viable alternative. A recent development is AI-based predictions of retinal function with the use of multimodal imaging modalities. So far, AI-based structure-function correlations have been applied for the inferring of conventional visual field and FCP data, BCVA, vision-related quality-of-life, and electroretinogram (ERG) characteristics.

This review summarises the progress in this field, compares goodness of fit measures and AI algorithms utilised while focusing on possible implementations in AMD patients.

## AI based structure-function correlation in visual fields

Although the focus of this review is on AMD relevant diagnostic tools, it is important to note that algorithms for inference of function have a long-standing history in glaucoma detection. First automated programs to diagnose visual field deficits date back to the 1980s and some studies now deploy OCT imaging to guide decision making [19, 20]. Newer studies, like Christopher et al. deploy large data sets of almost 10,000 visual field/OCT pairs from over a 1000 participants to train DL algorithms and are able to estimate the mean deviation of the visual field with an accuracy of 2.5 dB (*R*^2^ 0.7) [21]. Inference of sectoral visual field loss varied between high accuracy in the inferior-nasal sector (*R*^2^ 0.6) and low accuracy in both the central (*R*^2^ 0.15) and temporal (*R*^2^ 0.12) sectors. This algorithm was trained with retinal nerve fibre layer (RNFL) thickness maps and the results show that accuracy was lowest in areas with a physiological thin RNFL layer (temporal sector) and highest in areas with physiological thick RNFL layer (inferior-nasal sector) [22]. This is in accordance with the notion that pathological RNFL layer thinning is harder to discriminate in areas of decreased physiological thickness. Overall, progress on visual field Inferences will likely provide deeper insights into structural-functional correlations when large data sets may become available.

## AI based structure-function correlation in electroretinogram

In ABCA4-related retinopathy, a recent study predicted ERG Results through OCT layer thickness with an accuracy of 97.47 ± 2.03% [23]. The most relevant OCT-based imaging features in the applied machine learning approach were the outer nuclear layer (ONL) and the inner- and outer segments (IS/OS). The high impact of the ONL and IS/OS layers is biologically plausible as they represent parts of rod and cone photoreceptors. Notably, prediction accuracies may not necessarily translate to AMD as the ABCA4-related retinopathy cohort is a hereditary, monogenetic disease with rather well-defined ERG changes, while ERG findings in AMD are known to be much less specific. However, AI-based structure-function correlations in AMD subjects also demonstrated biological plausibility, showing that ONL thickness changes had the highest predictor importance for functional deficits that can be detected by other functional tests, including FCP (FCP; see below) [24–27].

## AI-based structure-function correlation in BCVA

In the context of AMD, inference of BCVA from OCT images has been proposed predominantly for macular neovascularisation (Table 1) [23, 27–31]. During anti-VEGF treatment, retinal imaging plays a pivotal role in disease management, with patients being regularly monitored by OCT. Beyond qualitative interpretation by the human eye, the automated analysis of these extensive amounts of imaging data may be particularly useful for estimation of BCVA, in addition to routine BCVA measurements, potentially sparing time and allowing for a more consistent assessment.

**Table 1:** AI-based structure-function in best-corrected visual acuity (BCVA).

Author/Ref.	Title	Disease	Technique	Prediction	Outcome measure	Outcome
Rohm et al. [27]	Predicting visual acuity by using machine learning in patients treated for neovascular age-related macular degeneration	Neovascular age-related macular degeneration	• Five different machine-learning algorithms • Best performance by Lasso regression	• logMAR visual acuity after 3- and 12 months	• Mean Absolute Error (MAE) • Root Mean Sqaured Error (RMSE)	• 3 Months = MAE: 0.11–014/RMSE: 0.18–0.2 • 12 Months = MAE: 0.16–0.2/RMSE: 0.2–0.22
Schmidt-Erfurth et al. [28]	Machine learning to analyze the prognostic value of current imaging biomarkers in neovascular age-related macular degeneration	Neovascular age-related macular degeneration	• Random forest	• BCVA at Baseline and 3 months follow-up	• Accuracy (R^2^)	• *R*^2^ = 0.21 baseline • *R*^2^ = 0.70 3 months
Gerenda et al. [29]	Computational image analysis for prognosis determination in DME	Diabetic macular edema	• Random forest	• BCVA at Baseline and 1-year follow-up	• Accuracy (R^2^)	• *R*^2^ = 0.21 baseline • *R*^2^ = 0.23 1 year
Aslam et al. [30]	Use of a neural net to model the impact of optical coherence tomography abnormalities on vision in age-related macular degeneration	Neovascular age-related macular degeneration	• Scaled conjugate gradient backpropagation (supervised learning)	• BCVA	• Root Mean Sqaured Error (RMSE)	• 8.21 Letters
Pfau et al. [31]	Artificial intelligence in ophthalmology: guideline for physicians for the critical evaluation of studies	Neovascular age-related macular degeneration	• Nested cross validation	• BCVA (LogMAR)	• MAE	• 0.142
Müller et al. [23]	Prediction of function in ABCA4-related retinopathy using ensemble machine learning	ABCA4-related Retinopathy	• Ensemble machine learning algorithms • Three models (a) Retinal layer (b) All structural data (c) demographic data	• BCVA • Divided into four categories from no to severe impairment	• Area under the curve (ROC)	(a) 88.64–92.25% (b) 90.23–93.68% (c) 87.26–91.44%
Sumaroka et al. [45]	Foveal therapy in blue cone monochromacy: predictions of visual potential from artificial intelligence	Blue Cone Monochromacy	• Random forest (a) Layer thickness (b) Reflectivity	• BCVA	• Root mean squared error (RMSE)	(a) 0.159 (b) 0.167

Until today, goodness of fit measures varied between studies for inference of BCVA based on imaging using AI tools. Two studies reported similar accuracies with a mean absolute error (MAE) of 0.11–0.14 LogMAR and 0.14 LogMAR. Another study documented their accuracy with a Root Mean Squared Error (RMSE) of 8.21 letters. The MAE, as the mean of the absolute values of the individual prediction errors, is an easy interpretable evaluation metric to judge the accuracy of regression models. For an exemplary patient with an BCVA of 0.3 LogMAR (Snellen 6/12), the inferred BCVA would be on average between 0.19 and 0.41 logMAR (~6/9–6/15). In some cases, when not only the average error but also the outliers are of interest, it may be helpful to indicate other evaluation metrics like the RMSE. The RMSE indicates the size of the squared error. As a result, larger errors have a disproportionately larger effect on the RMSE.

Rather than inferring function in a cross-sectional manner, another interesting aspect is to predict BCVA in the future, based on data available at baseline. This prediction might be particularly helpful to better estimate possible treatment effects. As one may expect, studies have reported lower accuracy in predicting BCVA as compared to the inference of BCVA based on imaging data from the same visit. By comparing five different ML algorithms, Rohm et al. [27] reported that cross-validated LASSO regression achieved most precise results with 0.14 logMAR RMSE (equals 7 letters) for the short-term (90 days from baseline) and 0.23 logMAR RMSE (equals 11.5 letters) for long-term predictions. This means that if an exemplary patient would have a measured BCVA of 0.3 logMAR (Snellen 6/12) at the 3 months follow-up visit, the model based on multimodal imaging at baseline would have predicted the BCVA being on average between 0.16 and 0.44 logMAR (~6/9–6/15). This study further demonstrated that not always the most complex AI algorithm achieved most accurate predictions. LASSO regression, the most accurate algorithm in this study, builds on linear regression and is therefore easily computable and easy for the non-AI specialist to interpret.

These algorithms could serve to inform the patient over their individual disease progression and give a prognosis of their driving capability. It should be considered that the 95% limits of agreement (LoA) of repeatability for tested BCVA is already about ±0.1 logMAR under perfect conditions for healthy subjects [32, 33]. Even with improved accuracy of the algorithm, it is important to note that inferred BCVA faces the same challenges and limitations as measured BCVA. These are specifically due to the ceiling effect (given the limited by the retinal peak cone density) and the relative focus on foveal (and/or para-foveal) function.

## AI-based structure-function correlation in patient-reported outcome measures

The European Medicines Agency and the Food and Drug Administration increasingly demand the employment of PROM as functional endpoints in clinical trials. During the Phase 2 Mahalo Study for GA, the 25-Item National Eye Institute Visual Function Questionnaire (NEI VFQ-25) has demonstrated to be a valid and reliable measure of patients Vision-Related Quality-of-Life (VRQoL) [34]. A recent study applied predictive modelling (LASSO regression) on both functional and structural biomarkers to project NEI VFQ-25 VRQoL for GA patients [35]. Interestingly, they found that VRQoL predominantly depended on the better eye. Structural biomarkers only explained up to 22% of variability but in combination with functional parameters like LLVA achieved excellent results in predicting VRQoL. These observations may be used for modelling of function in the clinical trial setting. In early phase clinical trials, a common approach is to rather test a new intervention in the worse as compared to the better eye because of ethical considerations, particularly the uncertainty of the risk profile of a new intervention. In this context, the authors suggested to extract the information from treating the worse eye to infer the expected effect on VRQoL in the better seeing eye [35].

## AI-based structure-function correlation in fundus-controlled perimetry

**Fig. 1:**
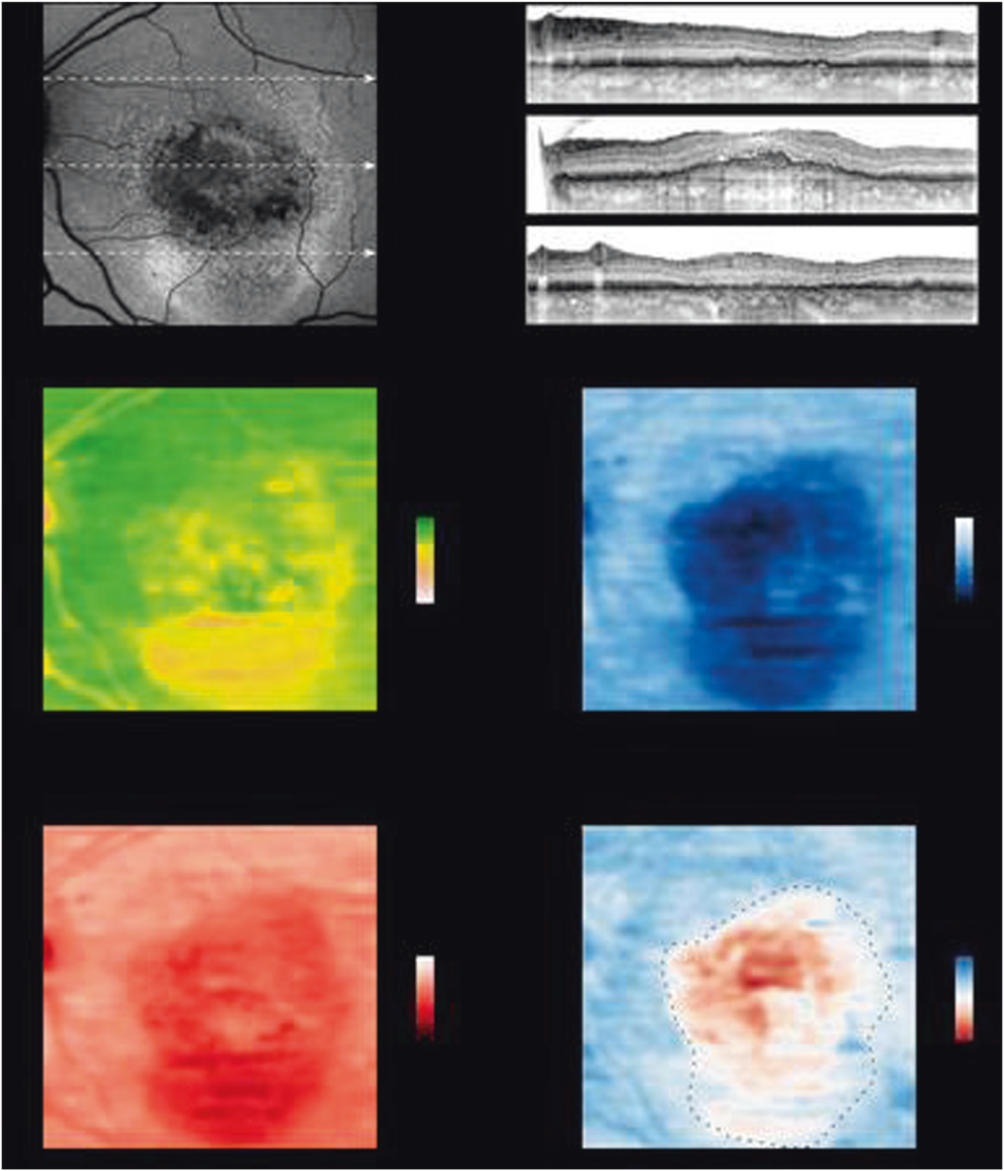
Inferred sensitivity mapping. Based on the fundus autofluorescence (FAF), infrared reflection (IR, not shown) and spectral-domain optical coherence tomography (SD-OCT), mesopic as well as dark-adapted (DA) cyan and DA red sensitivity may be reliably inferred and topographically mapped. The arrows in the FAF image indicate the position of the SD-OCT B-scans. Multiple lines of evidence further support the accuracy of the inference. For all three types of testing, angioscotoma are adequately predicted. Further, the central rod-free zone is also correctly inferred as indicated by the marked cyan-red sensitivity difference at the fovea (eccentricity of 0°, middle B-scan). Regions exhibiting increased FAF and absence of photoreceptor outer and inner-segments (upper and lower SD-OCT scan) show reduced function for all three types of testing. Yet globally the degree of DA cyan dysfunction appears to exceed the degree of DA red dysfunction. Please note, that the inferred cyan-red sensitivity difference in the region of severe cone dysfunction (delimited by the dashed line) is an underestimation of the true cyan-red sensitivity difference due to the floor effects of the perimetry device used in this study that are inevitable reflected by the models. (Reprinted from von der Emde et. al: Artificial intelligence for morphology-based function prediction in neovascular age-related macular degeneration; Scientific reports 9:1132; published [2019] Springer Nature).

Beyond BCVA which is limited to the assessment of foveal function, FCP (also termed microperimetry) can detect impairment throughout the central retina while correcting for fixation losses [36]. At the same time, FCP is time-consuming and burdensome for both patients and medical health care professionals. A recent development of FCP is the ability to test two-colour dark-adapted (DA) sensitivity. This setup allows to confirm previous reports, based on histopathology and general clinical assessment, that rod loss exceeds and precedes cone loss in eyes with AMD. FCP may allow for more precise assessment in the clinical setting as compared to more general clinical tests and therefore excel in detecting earliest changes in AMD. Using AI, early data indeed show that the precise probing of rod and cone function opens the door for a more detailed structure-function correlations between microstructural changes seen by multimodal imaging and retinal sensitivity as measured by FCP [37–40]. In this context, one interesting observation is that specific lesion components exhibit a distinct effect on cone (e.g. cuticular drusen with central, pseudo-vitelliform detachment), while other do on rod (e.g. reticular drusen; macular oedema) dysfunction [41, 42]. In neovascular late-stage AMD, FCP revealed a characteristic functional response to pigment epithelial detachments, subretinal- and intraretinal fluid [43]. For example, subretinal fluid appeared to affect rod function to a greater degree than cone function. Intraretinal fluid seemed to impair both cone and rod function to a similar extent. Participants with a shallow pigment epithelial detachment exhibited relative preserved cone and rod function. Given that structure and function are so closely intertwined, AI-based inference of retinal sensitivity through multimodal imaging (herein termed inferred sensitivity) would be tremendously advantageous beyond just replacing a burdensome psychophysical examination (Table 2). Inferred sensitivity has the potential to probe larger areas of the retina while still providing a high spatial resolution (Fig. 1). Additionally, with inferred sensitivity patients who are too frail for FCP examinations or have difficulty fixating could now partake in interventional studies. Finally, image acquisition for inferred sensitivity can be performed in centres without the expertise for psychophysical testing. So far inferred sensitivity has been explored in macular telangiectasia type 2, Leber congenital amaurosis (LCA), blue cone monochromasy, pseudoxanthoma elasticum as well as MNV and GA secondary to AMD, demonstrating that mesopic sensitivity can be inferred with an accuracy of 3.36–4.64 decibel (dB) MAE cross those diseases [24, 25, 44–47].

**Table 2:** AI-based structure-function in fundus-controlled perimetry (FCP).

Author/Ref.	Title	Disease	Technique	Prediction	Outcome measure	Outcome
Kihara et al. [44]	Estimating retinal sensitivity using optical coherence tomography with deep-learning algorithms in macular telangiectasia type 2	Macular telangiectasia type 2	1. Million-variable deep-learning model (convolutional neural network) 2. Letnet modell 3. Linear regression	• Fundus-controlled perimetry (FCP) (a) Mesopic	• Mean absolute error (MAE)	1. 3.36 dB 2. 3.66 dB 3. 4.51 dB
von der Emde and Pfau et al. [25]	Artificial intelligence for morphology-based function prediction in neovascular age-related macular degeneration	Neovascular age-related macular degeneration	• Random forest (LOO-CV) • Two scenarios 1. Without FCP 2. With FCP data	• Fundus-controlled perimetry (FCP): (a) Mesopic (b) DA Cyan (c) DA Red	• Mean absolute error (MAE) • Root mean squared error (RMSE)	• Scenario 1 • 3.94 dB (a) 4.89 dB (b) 4.05 dB (c) • Scenario 2 • 2.8 dB (a) 3.7 dB (b) 2.85 dB (c)
Pfau et al. [24]	Determinants of cone- and rod function in geographic atrophy: AI-based structure-function correlation	geographic atrophy secondary to age-related macular degeneration	• Random forest (LOO-CV) • Two scenarios 1. Without FCP 2. With FCP data	• Fundus-controlled perimetry (FCP): (a) mesopic (b) DA Cyan (c) DA Red	• Mean absolute error (MAE) • Root mean squared error (RMSE)	• Scenario 1 • 4.64 dB (a) 4.89 dB (b) 4.4 dB (c) • Scenario 2 • 2.89 dB (a) 2.86 dB (b) 2.77 dB (c)
Sumaroka et al. [46]	Treatment potential for macular cone vision in leber congenital amaurosis due to CEP290 or NPHP5 Mutations: predictions from artificial intelligence	Retinitis pigmentosa (RP; training) Congenital Amaurosis (LCA; prediction)	• Random forest (LOO-CV) • Two models 1. Thickness and eccentricity 2. Reflectivity	• Dark-adapted static perimetry (a) DA cyan (b) DA red	• 95th Percentile limits of agreement (LOA)	RP 1. 9.6 dB (a) 8.8 dB (b) 2. 11.9 dB (a) 10.8 dB (b) LCA 1. 4.6–17.6 dB
Sumaroka et al. [45]	Foveal therapy in blue cone monochromacy: predictions of visual potential from artificial intelligence	Blue Cone Monochromacy	• Random forest (a) Layer thickness (b) reflectivity	• Fundus-controlled Perimetry (a) Mesopic	• Root mean squared error (RMSE)	(a) 2.91 dB (b) 2.69 dB
Heß et al. [47]	Mesopic and scotopic light sensitivity and its microstructural correlates in pseudoxanthoma elasticum	Pseudoxanthoma Elasticum	• Random forest (LOO-CV)	• Fundus-controlled perimetry: (a) Mesopic (b) DA Cyan (c) DA Red	• Mean absolute error (MAE)	(a) 4.91 dB (b) 5.44 dB (c) 4.99 dB

DA cyan estimates achieved an accuracy of 4.89 dB MAE in late-stage AMD and 8.8 dB 95th percentile LoA in LCA. Similarly, accuracy in DA red testing was 4.05–4.64 dB MAE in late-stage AMD and 9.6 dB LoA in LCA. The results are further underscored by the fact that the 95% coefficient of repeatability for FCP testing was reported with (mean ± SD) 5.99 ± 1.55 dB for mesopic, 6.14 ± 2.19 dB for dark-adapted cyan and 6.06 ± 1.79 dB for dark-adapted red testing in MNV secondary to AMD (with similar results for GA) [6, 7]. Although evaluation metrics are not identical, this shows that the average error of inference based on multimodal imaging differs only marginally from the error resulting from repetitive testing (test-retest reliability) alone.

Studies using ML algorithms further analysed feature importance of inferred sensitivity and demonstrated that layer thicknesses proved more indicative for predictions than layer intensities [24, 25, 45–47]. Hereby, the results of the analysis of retinal thickness seemed biological plausible, as ONL thickness proofed to be the most important imaging feature in all types of testing and cross disease stage. Specifically, a thinning of the ONL (indicative of outer retinal atrophy) led to a decrease in sensitivity whereas pathological ONL thickening only negatively affected dark-adapted cyan and red sensitivity [24, 25].

In our studies in late-stage AMD patients, we further tried to boost results by feeding the algorithm the results from a short perimetry testing, since we hypothesised that factors not resolved by OCT imaging (e.g. lenticular opacification) may influence retinal light sensitivity [24, 25]. Indeed, addition of some functional data (in form of a subset of the perimetry results) markedly reduced the MAE up to 1.54 dB in mesopic and DA cyan and red testing [24, 25]. Although not as pronounced an alternative approach using ‘Patient reliability indices’ (e.g. False-positive response rate during FCP testing) also markedly reduced the MAE. These approaches in the context of an interventional study could improve accuracies of inferred sensitivity. Moreover, with additional short perimetry testing potential adverse effects of the agents (e.g. RNFL layer thinning) that are not represented in the training set could still be detected. In summary, we consider inferred sensitivity to be a potential surrogate functional endpoint and a valuable tool for future interventional studies.

## Limitations

Comparison of results among available studies is hampered by non-uniform use of goodness of fit measures. For example, the MAE is easily interpretable but may simulate better results than RMSE as it scrutinises outliers more harshly. Therefore, we suggest that studies reach consensus on measures of goodness of fit or to report additional measures for comparison. Another limitation is the cross-sectional study design of most AI-based function prediction studies so far. To safely utilise AI-based predictive modelling in future interventional studies, the accuracy of longitudinal models needs to be verified. Additionally, most studies were performed with a limited number of participants. This could potentially lead to underrepresenting less frequent manifestations in the training set. In AMD for example, these could be subretinal drusenoid deposits in intermediate AMD, retinal angiomatous proliferation in MNV secondary to AMD or the diffuse-trickling phenotype in GA secondary to AMD.

## Outlook

**Fig. 2:**
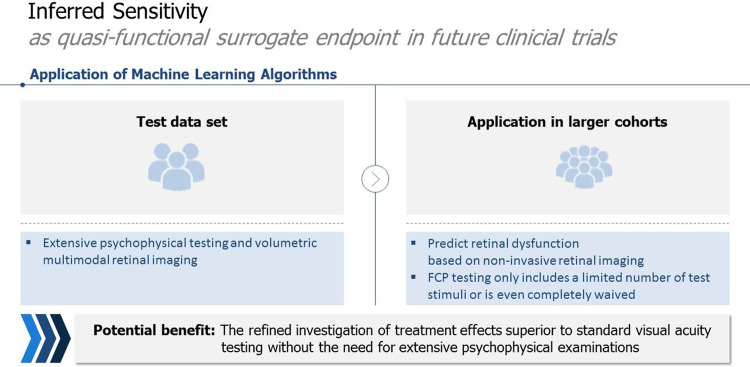
Inferred sensitivity. Possible quasi-functional surrogate endpoint in future clinical trial.

This review evaluated the current literature on AI-based function inference on a plethora of different psychophysical examinations. In retinal diseases other than AMD, AI-based function inferences serve to accurately forecast visual fields, ERG, BCVA and FCP examinations. In AMD, AI-based function inferences can compute BCVA, PROM and FCP results. We established the term ‘inferred sensitivity’ for multimodal imaging-based estimation of FCP results. Accuracy of inferred sensitivity can be improved by adding short FCP examinations in a subset of patients (Fig. 2). Inferred sensitivity of two-colour DA FCP can also estimate rod function and detect earliest visual impairment in AMD patients. Therefore, we consider inferred sensitivity to be a quasi-functional surrogate endpoint.
